# Trends of care patterns and outcomes of very low birth weight infants (≤1500 g) born after 27 weeks of gestation in South Wales, UK

**DOI:** 10.1038/s41372-025-02431-w

**Published:** 2025-09-18

**Authors:** Lieve Boel, Mirain Davies, Nitin Goel, Ian Paul Morris, Sujoy Banerjee, Vasiliki Makri, Chuen Poon, Anitha James, Mallinath Chakraborty

**Affiliations:** 1 Regional Neonatal Intensive Care Unit, University Hospital of Wales, Cardiff, UK; 2 School of Medicine, Cardiff University, Cardiff, UK; 3 Neonatal Unit, Singleton Hospital, Swansea, UK; 4 Neonatal Unit, Grange University Hospital, Cwmbran, UK

**Keywords:** Outcomes research, Epidemiology

## Abstract

**Objective:**

To analyse trends of outcomes of very low birth weight (VLBW) infants born after 27 weeks of gestation over 15 years in South Wales, UK.

**Study design:**

Trends of clinical outcomes were analysed by deriving multivariable logistic regression models and presented as odds ratios (aOR) with 95% confidence intervals (95% CI). A p-value of <0.05 was considered to be statistically significant.

**Results:**

Between 2007 and 2021, 2321 infants were included in the cohort. There was a decline in the incidence of mortality (aOR 0.941; 95% CI 0.895, 0.988), severe brain injury (0.937; 0.893, 0.982), necrotising enterocolitis (0.911; 0.862, 0.964) and sepsis (0.949; 0.920, 0.978). At birth, odds of mechanical ventilation (0.909; 0.888, 0.930) & receipt of surfactant (0.920; 0.899, 0.942), and mechanical ventilation after admission (0.940; 0.919, 0.961) were significantly reduced. Analysis of a subgroup of 1797 preterm infants born before 32 weeks suggested significant improvement in all major outcomes studied.

**Discussion:**

Trends of care patterns and outcomes improved over time in this cohort of VLBW infants in South Wales, especially preterm infants below 32 weeks of gestation.

## Introduction

Prematurity (birth before 37 completed weeks of gestation) remains a major risk factor for morbidity in the neonatal period. Although the trends in the incidence of prematurity in Europe have been stable [1] and survival is improving [2], prematurity remains an important factor for mortality in the newborn period [3]. While gestation at birth is a primary and dominant factor contributing to neonatal morbidity and mortality, birthweight has also been identified as an independent factor for these adverse outcomes [4, 5].

Birth weight is an objective and easily measurable variable for newborn infants, available commonly even in healthcare systems where accurate gestational assessment is limited, including infants who are preterm and/or who have not grown well in utero. A minority of newborn infants have very low birth weight (VLBW, ≤1500 g); in Wales, 242 of the 28,296 live births in 2022 (0.86%) had a birth weight of less than 1500 g (Office for National Statistics, UK; accessed March 2025). Most of these infants are appropriately grown for their gestation but born preterm, where a very low birth weight is expected (appropriate for gestational age, AGA). However, some infants have birth weights lower than expected for their gestational age and are referred to as small for gestational age (SGA). Outcomes of preterm SGA infants are not only associated with prematurity but also with the additional risks from their very low birth weight [6].

Survival and morbidity data are a valuable resource for teams managing these infants clinically to support counselling expectant parents and identifying areas for improvement. To our knowledge, only limited data are available in the published literature for this specific group of neonates. The majority of the complications of prematurity are concentrated in extreme preterm infants born before 28 weeks of gestation [2]. To understand the specific outcomes of other VLBW populations, we have separated and presented results from a cohort of infants who were born after 27 weeks of gestation (results from the extreme preterm cohort will be reported separately).

In South Wales, which is a geographically defined area in the UK, three tertiary neonatal units care for most infants who are born with a very low birth weight (≤1500 g). Data for these infants are quality assured and uploaded by experienced clinicians to the Vermont Oxford Network (https://public.vtoxford.org/) database for benchmarking purposes. In this paper, we report the temporal trends of mortality, morbidity and care practices in the South Wales Neonatal Network for VLBW infants who were born after 27 weeks of gestation with very low birth weight (≤1500 g) over 15 years (2007–2021).

## Methods

### Setting

South Wales is home to 75% of the population of Wales, with England as its eastern border and mid-Wales forming the northern border. The estimated birth rate in South Wales is around 25,000 per year [7]. Neonatal care for the population is centralised and served by three tertiary neonatal intensive care units (NICUs) that offer a full range of medical care for preterm infants; one of these units also serves as a regional centre for neonatal surgery and other subspecialty services. The care for very low birth weight (VLBW) infants is variable and has changed over time. Infants needing intensive care are either born at one of the tertiary centres or transferred into these centres soon after birth. Arrangements for infants needing high-dependency care have changed over time; since the mid-2010s, it has been agreed that infants born before 32 weeks or with a birthweight ≤1500 grams should be transferred to one of the tertiary units for initial care. Six local neonatal units provide high-dependency (limited) and special care for the more mature preterm infants closer to home, either from birth or following repatriation from the tertiary units; more recently, only three of these units offer care for neonates.

### Study population

The study cohort included all infants born alive after 27 weeks of gestation (≥28^+0^ completed weeks of gestation) with a birth weight of ≤1500 g between 1st January 2007 and 31st December 2021 at one of the three tertiary neonatal units in South Wales (inborn infants). Infants born at any of the local neonatal units and transferred into one of the three tertiary units were also included in the cohort (outborn infants). No exclusion criteria were applied; however, infants who were born alive at local neonatal units but died before transfer to a tertiary unit were not included in the cohort. As a variable known to affect outcomes, statistical analysis accounted for the differences in the place of birth [8, 9].

### Data collection

Since 2007, all three tertiary neonatal units have contributed data for benchmarking purposes to the Vermont Oxford Network (VON) for all infants born at <30 weeks of gestation or with birth weight ≤1500 g. Data for the VON database was collected and verified by a designated senior clinician in each unit, ensuring consistency and high quality. This study included prospectively collected anonymised data from all three tertiary neonatal units over a 15-year study period for eligible infants.

Raw data included maternal and neonatal characteristics, resuscitation practices, early clinical management, and clinical outcomes, including death and major morbidities. The data was collected for each infant for their first admission to the NICU until death or transfer to another non-VON centre or discharge home from the tertiary unit. Standard VON definitions were used for major morbidities (https://help.vtoxford.org/nightingale/help/#4662.htm) and are described in detail in Supplementary Table 1.

### Exposure variable, other variables, and outcomes

The primary exposure variable was the year of birth, which was treated as a continuous variable in all statistical analyses.

The following covariates (independent variables) were used for adjusted analyses as appropriate:

1. Gestation (continuous variable used as a decimal by adding any additional days to completed weeks)
2. Sex (binary variable)
3. Small for gestational age (SGA), defined as a gestation-specific birthweight <10th centile or z-score <1.282
4. Year of birth (continuous variable)
5. Place of birth, i.e. inborn or outborn (binary variable)
6. Non-white ethnicity (self-declared by family)
7. Exposure to antenatal steroids (binary variable)
8. Multiple births (binary variable)
9. Vaginal delivery (binary variable)
10. Admission temperature (continuous variable)

The co-primary outcomes (dependent variables) included the following major outcomes:

1. Mortality after live birth and after admission to NICU
2. Severe brain injury, defined as a composite of severe intraventricular haemorrhage (IVH) grade III-IV [10] OR periventricular leukomalacia, detected by cranial ultrasound (CUSS) abnormalities
3. Treatment for retinopathy of prematurity (ROP), including laser therapy and/or anti-VEGF injections
4. Bronchopulmonary dysplasia (BPD) at 36 weeks is defined as a requirement for mechanical ventilation, non-invasive ventilation, or supplemental oxygen at 36 weeks post-menstrual age [11]
5. Necrotising enterocolitis (NEC) is defined as Bell stage II-III [12]
6. Sepsis during the stay, defined as a culture-positive episode with bacterial or fungal organisms in bodily fluid that is considered sterile (blood, cerebrospinal fluid)

Secondary outcomes included key changes in respiratory support practice, including the use of surfactant, the use of non-invasive respiratory support and the use of mechanical ventilation.

### Statistical analysis

Statistical analysis was undertaken in SPSS version 29 (IBM Corporation, New York, USA). Initial analysis was conducted by dividing the whole cohort into three pragmatic epochs: 2007–2011 (epoch 1), 2012–2016 (epoch 2) and 2017–2021 (epoch 3). Missing or unavailable data were appropriately coded and automatically excluded from analysis at all stages. These are presented in Supplementary Table 2. All data were analysed on a per-infant basis and not corrected for multiple gestations (twins, triplets, etc.) as these were not identifiable. Continuous data (gestation, birthweight, birth head-circumference and admission temperature) are presented as medians with inter-quartile range (IQR) while categorical data are presented as proportions (percentages). Epochs were compared using the Kruskal-Wallis test for continuous variables and the Chi-square test for categorical variables.

Trends were analysed by deriving multivariable logistic regression models in blocks with the outcome of interest (co-primary and secondary outcomes, as stated above) as the dependent variable. The combined cohort (2007–2021) was used for the primary analyses; a subgroup analysis was also conducted for very preterm infants (born before 32 weeks of gestation) in the cohort. For each outcome, univariable analysis was conducted with all the covariates as above (except the year of birth) to identify significant associations, defined as *p* ≤ 0.1 on statistical testing (Mann–Whitney test for continuous variables and Chi-square test for categorical variables). Results of the univariable outcomes with statistical significance are presented in Supplementary Table 3. Variables identified at this stage were subsequently used for adjusted multivariable analysis.

1. In the first block, the dependent variable was modelled against the year of birth as an independent (continuous) variable and presented as an unadjusted Odds Ratio (OR).
2. In the second block, the regression analyses were adjusted for confounders by including covariates identified in the univariable analysis as significant associations and presented as adjusted OR.

A *p* value of <0.05 was considered statistically significant. All uses of the word “significant” in the results section refer to statistical significance only.

### Ethics

As this analysis was conducted on non-identifiable retrospective information that was part of routine data collection for clinical purposes, it was excluded from specific ethical approval as per the general guidance on the NHS Health Research Authority guidance tool for ethics approval. There was also no direct involvement of patients or their parents/guardians, as none were identifiable. A data-sharing agreement was signed and approved by the NHS Research and Development Department at each site. Categorical variables with *n* < 5 have been suppressed in tables to prevent accidental identification of individuals.

## Results

### Study population characteristics

In the 15 years between 2007 and 2021, 2321 infants (birthweight ≤1500 g and >27 weeks of gestation) were born or cared for in the three tertiary neonatal units in South Wales. This accounted for 95% of the infants fulfilling these criteria who were born in a hospital in South Wales (5% were born in a smaller hospital in the region and received their care in the same hospital without being transferred), which was estimated by comparing data between 2011 and 2020 from the Wales Maternity and Neonatal Network database. In the whole cohort,1797 (77.4%) infants were born before 32 weeks of gestation (very preterm); they were included in a subgroup analysis. Detailed demographic data on maternal and neonatal characteristics in each epoch and the overall cohort are presented in Table 1. Of note, there was a steady improvement in exposure to antenatal steroids, exposure to magnesium sulphate and the admission temperature of infants over the study period. A reduction in major birth defects and the incidence of chorioamnionitis was also noted during the 15 years.

**Table 1:** Infant and maternal demographics.

Demographics	2007–2011: 5-year incidence *n* (%age of available data within epoch)	2012–2016: 5-year incidence *n* (%age of available data within epoch)	2017–2021: 5-year incidence *n* (%age of available data within epoch)	Overall total (2007–2021)	*p* value
Infant					
Total infants	748	765	808	2321	–
Very preterm infants (< 32 weeks)	571 (76.3)	604 (79.0)	622 (77.0)	1797 (77.4)	**–**
Term infants (≥ 37 weeks)	*	*	*	4	
Total admitted infants	744	763	805	2312	–
Outborn	162 (21.7)	200 (26.1)	164 (20.3)	526 (22.7)	0.016
Birth gestation (weeks + days), median (IQR)	30.14 (2.71)	30.14 (2.5)	30.14 (2.57)	30.14 (2.57)	0.95
Birth weight (g), median (IQR)	1280 (320)	1280 (295)	1260 (285)	1278 (303)	0.418
Small for gestational age (less than 10th centile)	292 (39)	292 (38.2)	308 (38.1)	892 (38.4)	0.918
Appropriate for gestational age (between 10^th^ and 90^th^ centile)	423 (56.6)	447 (58.4)	475 (58.8)	1345 (57.9)	0.636
Large for gestational age (greater than 90^th^ centile)	33 (4.4)	26 (3.4)	25 (3.1)	84 (3.6)	0.351
Head circumference at birth (cm), median (IQR)	27.3 (2.3)	27.4 (2.3)	27.4 (2)	27.3 (2.3)	0.782
Admission temperature at birth (°C), median (IQR)	36.7 (0.6)	36.7 (0.6)	36.9 (0.5)	36.8 (0.6)	<0.001
Male	382 (51.1)	414 (54.1)	399 (49.4)	1195 (51.5)	0.165
Multiple births	224 (29.9)	216 (28.2)	219 (27.1)	659 (28.4)	0.459
Major birth defect	67 (9)	42 (5.5)	47 (5.8)	156 (6.7)	0.012
Maternal					
Non-white	76 (10.2)	79 (9.4)	66 (7.6)	221 (9)	0.201
Prenatal care	739 (99.1)	754 (99)	792 (98)	2285 (98.7)	0.142
Chorioamnionitis (from 2008)	74 (12)	66 (8.8)	47 (6)	187 (8.7)	<0.001
Maternal hypertension, chronic or pregnancy-induced (from 2008)	147 (23.8)	186 (24.4)	183 (23)	516 (23.8)	0.811
Antenatal steroid exposure	663 (88.9)	679 (89.3)	750 (93.3)	2092 (90.6)	0.005
Vaginal delivery	232 (31)	215 (28.1)	216 (26.7)	663 (28.6)	0.165
Antenatal magnesium sulphate (from 2012)	N/A	49 (6.8)	436 (57.4)	485 (25.9)	<0.001

### Mortality and major morbidities (Table 2)

**Fig. 1:**
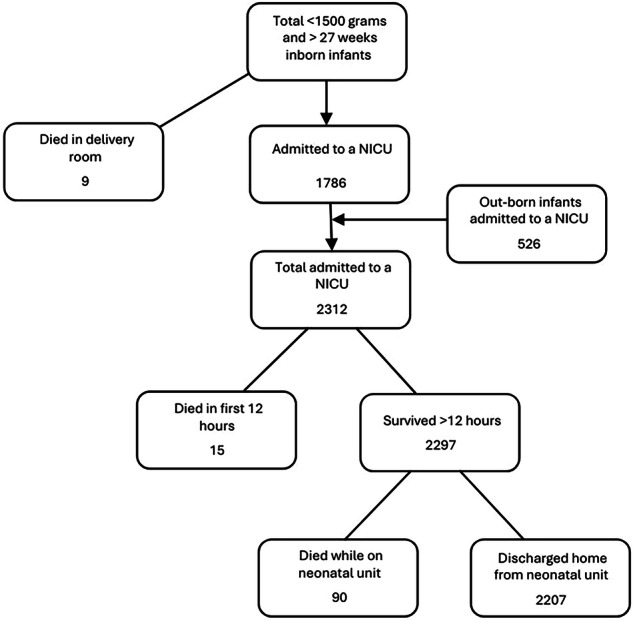
Summary of outcomes of included infants.

Figure 1 summarises the outcomes of all live births during the study period; a summary of mortality and morbidity outcomes is presented in Table 2. The majority of the infants who were born alive were admitted to a neonatal unit for further care; 9 infants (0.4%) died in the delivery room. Mortality of admitted infants steadily declined over the three epochs, as did any mortality after live birth (which includes delivery room deaths). Infants identified as SGA at birth had significantly increased odds of death. An increase in admission temperature reduced the odds of death significantly.

**Table 2:** Main outcomes over the study period.

Key outcomes	2007–2011: 5-year incidence *n* (%age of available data within epoch)	2012–2016: 5-year incidence *n* (%age of available data within epoch)	2017–2021: 5-year incidence *n* (%age of available data within epoch)	Overall total (2007–2021)	*p* value
Total infants	748	765	808	2321	–
Total admitted infants	744	763	805	2312	–
Delivery room deaths	*	*	*	9 (0.4)	0.691
Died after admission and before discharge	43 (5.8)	37 (4.8)	25 (3.1)	105 (4.5)	0.036
Any death after live birth	47 (6.3)	39 (5.1)	28 (3.5)	114 (4.9)	0.035
Severe IVH OR PVL (severe CUSS abnormalities)	44 (6.2)	37 (5)	27 (3.4)	108 (4.8)	0.042
ROP intervention (anti-VEGF^$^ or laser surgery)	10 (1.3)	6 (0.8)	4 (0.5)	20 (0.9)	0.194
Bronchopulmonary dysplasia (from 2011)	14 (17.9)	95 (24.2)	106 (24.7)	215 (23.9)	0.428
Medical treatment for PDA	32 (5.2)	25 (3.3)	14 (1.7)	71 (3.3)	0.001
Surgical treatment for PDA	6 (0.8)	1 (0.1)	1 (0.6)	8 (0.5)	0.160
Any treatment for PDA	36 (5.8)	25 (3.3)	15 (8.7)	76 (4.9)	0.004
Necrotising enterocolitis (NEC)	36 (4.8)	26 (3.4)	15 (1.9)	77 (3.3)	0.005
Any sepsis during stay	106 (14.8)	92 (12.5)	79 (10.2)	277 (12.4)	0.023
Any early-onset sepsis	8 (1.1)	16 (2.1)	5 (0.6)	29 (1.3)	0.027
Any late-onset sepsis	100 (14)	78 (10.6)	74 (9.5)	252 (11.3)	0.017

(* = numbers suppressed, ^$^anti-VEGF injections became available from 2012 onwards).

Over the three epochs, there was a decline in the incidence of severe cranial ultrasound abnormalities, treatment for PDA, and the incidence of necrotising enterocolitis and sepsis at any point during the stay. The overall incidence of any of these morbidities or mortality was significantly different from the extreme preterm cohort of the same population [2]. Apart from the temporal trend, gestation was identified as an independent variable for all the outcomes. Other independent variables that were statistically significant are shown in Table 3. Significant independent variables associated with increased odds of adverse outcomes included severe brain injury in the absence of antenatal steroid exposure, intervention for ROP, BPD and sepsis in infants delivered by Caesarean Section, as well as SGA status, outborn status and male sex increasing the odds of developing BPD.

**Table 3:** Trends of key outcomes by unadjusted and adjusted logistic regression models.

Outcomes	Unadjusted odds ratio (confidence intervals)	Adjusted odds ratio (confidence intervals)	Other Independent Variables, odds ratio (confidence intervals)
**All infants (n** = **2321)**			
Mortality	0.932 (0.889, 0.977)	0.941 (0.895, 0.988)	SGA: 2.784 (1.775, 4.366) Gestation: 0.726 (0.638, 0.827) Admission temperature: 0.727 (0.549, 0.962)
Severe brain injury (IVH grade III-IV OR PVL by CUSS)	0.933 (0.891, 0.977)	0.937 (0.893, 0.982)	No antenatal steroids: 3.210 (1.941, 5.309) Gestation: 0.661 (0.559, 0.781)
ROP intervention	0.897 (0.805, 1)	0.901 (0.808, 1.004)	C Section: 4.522 (1.041, 19.647) Gestation: 0.69 (0.506, 0.939)
BPD (from 2011)	1.03 (0.983, 1.08)	1.036 (0.985, 1.089)	Gestation: 0.681 (0.613, 0.757) SGA: 2.349 (1.597, 3.456) Out-born: 1.619 (1.032, 2.541) Male: 1.789 (1.279, 2.503) Vaginal delivery: 0.562 (0.368, 0.859)
NEC	0.912 (0.863, 0.964)	0.911 (0.862, 0.964)	Gestation: 0.761(0.653, 0.887)
Any sepsis during stay	0.952 (0.924, 0.981)	0.949 (0.920, 0.978)	C Section: 1.594 (1.179, 2.156) Gestation: 0.749 (0.689, 0.815)
Delivery room ventilation	0.926 (0.908, 0.945)	0.909 (0.888, 0.930)	SGA: 1.651 (1.300, 2.096) Multiple births: 0.788 (0.631, 0.984) No Antenatal steroids: 1.754 (1.231, 2.500) Gestation: 0.468 (0.432, 0.507)
Delivery room surfactant	0.936 (0.917, 0.955)	0.920 (0.899, 0.942)	SGA: 1.724 (1.353, 2.198) No Antenatal steroids: 1.635 (1.148, 2.329) Gestation: 0.443 (0.407, 0.482)
Ventilation after admission	0.955 (0.937, 0.974)	0.940 (0.919, 0.961)	SGA: 1.598 (1.260, 2.028) Males: 1.344 (1.109, 1.630) Outborn: 1.703 (1.346, 2.155) No Antenatal steroids: 1.764 (1.217, 2.558) Gestation: 0.501 (0.466, 0.540)
**Very preterm infants (n** = **1797)**			
Mortality	0.924 (0.878, 0.974)	0.935 (0.886, 0.987)	SGA: 2.693 (1.613, 4.497) Gestation: 0.655 (0.528, 0.813) Admission temperature: 0.682 (0.505, 0.922)
Severe brain injury (IVH grade III-IV OR PVL by CUSS)	0.923 (0.879, 0.969)	0.926 (0.880, 0.974)	SGA: 0.435 (0.226, 0.839) No antenatal steroids: 3.735 (2.186, 6.382) Gestation: 0.541 (0.432, 0.679)
ROP intervention	0.854 (0.753, 0.970)	0.865 (0.760, 0.985)	Gestation: 0.459 (0.271, 0.776)
BPD (from 2011)	0.883 (0.846, 0.921)	0.891 (0.851, 0.934)	SGA: 2.584 (1.706, 3.914) Male: 2.021 (1.406, 2.906) C Section: 1.841 (1.192, 2.842) Gestation: 0.547 (0.461, 0.649)
NEC	0.908 (0.856, 0.962)	0.911 (0.858, 0.967)	Gestation: 0.679 (0.534, 0.864) Non-white ethnicity: 2.490 (1.254, 4.945)
Any sepsis during stay	0.952 (0.922, 0.983)	0.956 (0.924, 0.988)	Gestation: 0.679 (0.592, 0.780) C Section: 1.601 (1.138, 2.251)
Delivery room ventilation	0.912 (0.891, 0.933)	0.900 (0.878, 0.923)	SGA: 1.383 (1.053, 1.816) Male: 1.296 (1.048, 1.603) No antenatal steroids: 1.730 (1.171, 2.556) C Section: 1.395 (1.098, 1.771) Gestation: 0.404, (0.360, 0.452)
Delivery room surfactant	0.918 (0.898, 0.939)	0.907 (0.884, 0.930)	SGA: 1.426 (1.081, 1.881) Male: 1.347 (1.086, 1.670) No antenatal steroids: 1.689 (1.143, 2.495) C Section: 1.478 (1.161, 1.882) Gestation: 0.378 (0.336, 0.424)
Ventilation after admission	0.937 (0.916, 0.959)	0.928 (0.905, 0.953)	SGA: 1.397 (1.055, 1.850) Outborn: 1.641 (1.257, 2.142) Male: 1.502 (1.207, 1.870) No antenatal steroids: 1.830 (1.187, 2.820) C Section: 1.817 (1.422, 2.321) Gestation: 0.423, (0.377, 0.475)

Sub-group analysis of the very preterm infants in our cohort demonstrated similar improvements in outcomes and care practices, with notable additional associations. Firstly, both ROP interventions and BPD showed statistically significant reduced odds over the study period, although these were not significant in the whole cohort. This meant that all outcomes and care patterns showed improving trends in the sub-group of infants. Association with other independent variables were similar, as shown in the whole cohort. This additional data is shown in Table 3.

### Changes in respiratory practice (Table 4)

Over the study period, the odds of mechanical ventilation and receipt of surfactant at birth in the delivery room significantly reduced (Table 4). A lack of exposure to antenatal steroids, lower gestation at birth and being identified as SGA at birth were independent predictors of both outcomes. After admission to the neonatal unit, the odds of mechanical ventilation also reduced significantly over the 15 years. In addition, male sex and being outborn were independent predictors of mechanical ventilation after admission.

**Table 4:** Changes in respiratory practice.

Key changes in practice	2007–2011: 5-year incidence *n* (%age of available data)	2012–2016: 5-year incidence *n* (%age of available data)	2017–2021: 5-year incidence *n* (%age of available data)	Overall total (2007–2021)	*p* value
Total infants	748	765	808	2321	–
Total admitted infants	744	763	805	2312	–
Surfactant during initial resuscitation	311 (41.7)	316 (41.3)	221 (27.4)	848 (36.6)	<0.001
Surfactant at any time (absolute)	415 (55.6)	420 (54.9)	413 (51.1)	1248 (53.8)	0.155
Any non-invasive ventilation during resuscitation (face-mask, CPAP)	498 (95.6)	673 (88.1)	761 (94.3)	1932 (92.4)	<0.001
Any non-invasive ventilation after resuscitation	662 (100)	706 (99.2)	777 (99.1)	2145 (99.4)	0.055
Any mechanical ventilation during resuscitation	336 (44.9)	319 (41.7)	231 (28.6)	886 (38.2)	<0.001
Any mechanical ventilation after initial resuscitation	439 (59)	451 (59.1)	384 (47.4)	1274 (55.1)	<0.001

## Discussion

In this retrospective database review, we report outcomes for 2321 VLBW infants born after 27 weeks of gestation and assessed trends over 15 years, divided into three 5-year epochs. Our results suggest that in this group of vulnerable infants cared for in the three tertiary units in South Wales, there was a significant reduction in mortality, severe brain injury, NEC, and sepsis during the study period, along with a non-significant increase in the incidence of BPD. One of the key changes in practice was the reduction of mechanical ventilation/surfactant use after birth, both during resuscitation and after admission to NICU.

Reports about this very specific population of preterm infants are sparse, and most include VLBW infants of all gestations, including those born at extreme preterm gestation (<28 weeks). The EPIPAGE study reported stratified data from a French cohort of preterm infants born at or before 34 weeks of gestation in 2011 and compared these to their cohort from 1997 [13]. In the 2011 cohort, 2509 infants were born between 28 and 31 weeks of gestation (median birthweights with IQR were >1500 g for infants ≥32 weeks of gestation). 2393 infants survived to discharge, suggesting a mortality rate of 4.6% which was similar to our cohort. Incidence of severe IVH was 3.1% (73/2351), PVL was 1.6% (39/2365), severe BPD was 3.1% (71/2322), severe ROP was 0.13% (3/2375), and severe NEC was 3.2% (76/2367). While the crude rates of severe IVH and NEC were comparable to our cohort, BPD was significantly more common in our cohort, likely because of the predominance of SGA infants. We have, by definition, measured moderate-severe BPD, contributing to a higher proportion of infants diagnosed with the condition. The predominance of SGA infants may also explain the slightly higher rates of severe ROP in our cohort (qualifying for intervention).

Vanhaesebrouck et al. [14] reported outcomes for 2037 VLBW infants from Belgium with a median gestational age of 29 weeks over 21 years (2000-2020). Similar to our study, the authors reported a steady decline in mortality rates and IVH grade III-IV, and a significant increase in the incidence of BPD, but no significant change in the incidence of NEC or sepsis was reported over their study period. This study differs from ours in several aspects: the cohort included extreme preterm infants, definitions of outcomes were more subjective (NEC was defined from grade I-IV and sepsis was defined by clinical criteria), and no trend analysis was reported by the authors.

Yang He et al. [15] studied 1750 VLBW Chinese infants (343 born at less than 28 weeks of gestation, 1032 at 28-32 weeks, 375 after 32 weeks) born between 2016-2021 and compared them with a retrospective cohort of 1146 VLBW infants born 2009-2015 (all infants included in this analysis). The authors published stratified results for infants with a birthweight between 1000 and 1500 g, which comes closest to our cohort of infants. Their incidence of mortality, severe brain injury, confirmed NEC and confirmed sepsis was comparable to our cohort. When compared with the retrospective cohort, the authors reported a significant reduction in the risk of mortality from 13.8 to 4.8% (RR 0.356, 95%CI 0.269–0.471). Of note, data definitions for most of the morbidity outcomes in the cohort were identical to VON definitions and were thus directly comparable with our outcomes.

Jiun Lee and colleagues [16] reported outcomes of 905 VLBW infants of all gestations in Singapore between 2015 and 2017 and compared them with a retrospective cohort from 2007–2008. There were reductions in the incidence of sepsis, but stable incidences of severe brain injury and NEC. While the data on BPD were incomplete, its components seemed to have increased between the two periods, as in our cohort. However, as their cohort included extreme preterm infants, these results are not directly comparable to our cohort.

In keeping with global trends, neonatal mortality rates in our cohort reduced significantly over time.

This was reflected in the literature, including the EPIPAGE cohort and the Chinese, Belgian and Singapore cohorts. While the populations were not homogeneous and perfectly comparable, there is likely to be variability in other factors that contribute to mortality. BPD is an outcome that is increasing over time both in our population, which includes a relatively large percentage of SGA infants, and in other VLBW populations [14–16]. It is well known that a lower gestational age and a lower birth weight are both associated with a higher risk for BPD [17, 18]. However, results for other morbidity outcomes are less uniform. We identified a decrease in ROP that needed intervention. This is in contrast with the increase in ROP in the Belgian [14] and Chinese VLBW population [15], although both of these cohorts include extreme preterm infants. The incidence of severe ROP was lower in the comparable infants in the EPIPAGE cohort [13], although that could be explained by a large proportion of SGA infants in our cohort. NEC incidence was also variable; stable over time in Belgium and Singapore [14, 16] but increasing in the Chinese [15] in VLBW populations, whilst a decrease was noted in our population, which did not include extreme preterm infants. Sepsis decreased over time in our population and almost halved over time in the cohort from Singapore [16], while there was a significant increase in the incidence of sepsis in the Chinese study [15]. The differences in morbidity trends could potentially be explained by different definitions used or the different study populations and settings, but more likely due to the absence of extreme preterm infants in our cohort. In addition, differences in perinatal optimisation, ventilation methods and general care of sick infants could be potential explanations for this observation.

In our cohort, logistic regression analysis identified gestation at birth (a unit increase of gestation at birth reduced the odds of mortality by 28%) and SGA (SGA status increased the odds of mortality by 2.8 times) as independent variables that contributed to most of the outcomes. In addition, as noted in multiple papers, lower admission temperature increased the odds of mortality [19–21]. Non-exposure to antenatal corticosteroids increased the odds of being ventilated in the delivery room and receiving surfactant, as well as needing mechanical ventilation after admission to NICU. Being born by Caesarean section increased the odds of ROP interventions and the incidence of sepsis after birth, although the reasons for these associations are not obvious.

We report an increase over time in the use of antenatal steroids and magnesium sulphate as reported in the Belgian and Singapore cohorts. This is most likely a reflection of the fact that the administration of antenatal steroids and magnesium sulphate to mothers at risk of preterm delivery has become standard practice over the last few decades. Respiratory care practices have evolved from mechanical ventilation to more non-invasive ventilation, intending to reduce BPD [22]. This trend is also reflected in our cohort, where we report a significant decrease in the use of mechanical ventilation and a significant increase in the use of non-invasive ventilation, both during stabilisation in the delivery room and after admission to the neonatal unit. We do report a significant decrease in the administration of surfactant during resuscitation at birth, but stable figures for surfactant administration during admission. This could be because less-invasive methods for surfactant administration, which have been recently introduced, are not commonly used during initial stabilisation but have gained popularity for use in the neonatal unit after admission without progressing to mechanical ventilation.

While we have identified improvements in outcomes, we have not been able to explore the causes of the improvements in our cohort. Both antenatal steroids [23] and exogenous surfactant replacement therapy [24, 25] have been standard practice in this cohort for several decades. More recently, based on the results of large randomised controlled trials [22], practice has recently shifted from mechanical ventilation towards more non-invasive respiratory support. As suggested by our results, changes in respiratory care patterns are likely to have contributed to overall improvements in neonatal care. Another key progress in neonatal care has been the introduction of delayed cord clamping, with significant survival benefits [26]. Unfortunately, this data was not available on the VON database during the study period. Additionally, nutritional care and growth of infants are also known to improve neonatal outcomes [27], but this data is not collected in the VON database. The improvements in outcomes are likely the result of a combination of proven interventions, both measured and unmeasured, and general refinements in neonatal care that have happened during the 15 years. Further studies to identify relevant factors that improve care and outcomes are recommended.

Our choice of cohort and analysis has several strengths. The data is derived from the VON database, which is entered by a senior clinician in each unit, increasing the likelihood of uniformity of interpretation. It spans 15 years, reflecting several changing practices in neonatal care (introduction of magnesium sulphate, use of CPAP at birth, delivery of surfactant by less invasive methods, etc). We have excluded the extreme preterm infants born before 28 weeks of gestation (reported separately) from this cohort, as most of the morbidity and mortality is concentrated in that group of infants; thus, our analysis provides trends of outcomes for VLBW infants specifically, including a significant proportion of SGA infants. However, some limitations inherent to this type of data analysis remain. A small number of infants who were born and cared for in the smaller neonatal units were not included in this analysis, as the data were not available. This includes infants who died in the delivery room and were never admitted to a neonatal unit. We have also used an absolute definition of SGA – birth weight lower than the 10^th^ centile for gestation. However, a small proportion of these infants may be “constitutionally small” due to various causes, like maternal stature or ethnicity. While adjusted foetal growth charts to account for maternal stature are used in clinical practice (https://perinatal.org.uk/GAP/Programme), this data is not recorded in the VON database and thus not available to us. This may have slightly overestimated the proportion of infants classified as SGA at birth.

In summary, we have described outcome data and trends over time for a very specific group of preterm infants, those with a birth weight of less than 1500 grams but with a gestational age of more than 27 weeks. The median gestational age was 30.14 weeks, and the median birth weight was 1278 grams in this cohort. This means they are more mature and have a higher birth weight, although still very low birth weight, than the extreme preterm infants in the cohort we reported on previously [2].

These differences could explain why the incidence of morbidity in this population is significantly lower for all outcomes described, as it is well-known and previously reported that morbidity is higher at lower gestational ages. This also accounts for the significantly lower mortality rate in this cohort (4.9%) than in our extreme preterm cohort (28.2%). As repeatedly identified, SGA status at birth remains a significant and independent risk factor for multiple major neonatal outcomes. As the causes of foetal growth restriction (FGR), the presumed precursor for SGA infants, are diverse [28], further research into reducing FGR and its causes could result in improving neonatal outcomes.

## Supplementary information

Supplementary Information

## Data Availability

All relevant data have been published with the manuscript; original data are not available due to restrictions on sharing.
