# Supplementary material for: Trends of care patterns and outcomes of very low birth weight infants (≤1500 g) born after 27 weeks of gestation in South Wales, UK
Source: J Perinatol. 2025 Sep 18;46(8):1506–14. doi: 10.1038/s41372-025-02431-w (PMC13473016; doi:10.1038/s41372-025-02431-w)
Supplement: Supplementary file 1 [file 41372_2025_2431_MOESM1_ESM.docx]

**Trends of Care Patterns and Outcomes of Very Low Birth Weight Infants (≤1500 gm) born after 27 weeks of gestation in South Wales, UK**

Lieve Boel, Mirain Davies, Nitin Goel, Ian Paul Morris, Sujoy Banerjee, Vasiliki Makri, Chuen Poon, Anitha James, Mallinath Chakraborty

**Supplementary Information**

| **Morbidity** | **Definition** |
| --- | --- |
| Bronchopulmonary dysplasia  (BPD) | need for supplemental oxygen, conventional ventilation or non-invasive ventilation at 36 weeks post-menstrual age (PMA)^1^ |
| Necrotising enterocolitis  (NEC) | stage II or III as defined by the modified Bell staging criteria^2^ |
| Severe intraventricular haemorrhage (IVH) | grade three or four in the Papile classification for IVH^3^ |
| Retinopathy of prematurity  (ROP) | defined by the International Classification of ROP^4^ |
| Severe ROP | stage three or higher on this classification scale |
| Early-onset sepsis (EOS) | a bacterial pathogen cultured on blood or cerebrospinal fluid before the third day of life |
| Late-onset sepsis (LOS) | a bacterial pathogen cultured on blood or cerebrospinal  fluid after the third day of life |

**Supplementary Table 1: Definitions of major morbidities used in the study.**

| **Demographics** | **2007-2011:**  **Epoch 1** | | **2012-2016:**  **Epoch 2** | | **2017-2021:**  **Epoch 3** | | **Overall total**  **(2007-2021)** | |
| --- | --- | --- | --- | --- | --- | --- | --- | --- |
| Total infants | 748 | | 765 | | 808 | | 2,321 | |
| Total admitted  infants | 744 | | 763 | | 805 | | 2,312 | |
|  | Missing  data (%age  from total  admitted infants) | N/A | Missing  data (%age  from total  admitted infants) | N/A | Missing  data (%age  from total  admitted infants) | N/A | Missing  data (%age  from the total admitted infants) | N/A |
| Outborn | 0 | 0 | 0 | 0 | 0 | 0 | 0 | 0 |
| Birth gestation (weeks + days), median (IQR) | 0 | 0 | 0 | 0 | 0 | 0 | 0 | 0 |
| Birth weight (g), median (IQR) | 0 | 0 | 0 | 0 | 0 | 0 | 0 | 0 |
| Head  circumference at birth (cm),  median (IQR) | 90 | 0 | 54 | 0 | 34 | 0 | 178 | 0 |

| Admission  temperature at birth (°C),  median (IQR) | 9 | 0 | 5 | 0 | 4 | 0 | 18 | 0 | |
| --- | --- | --- | --- | --- | --- | --- | --- | --- | --- |
| Male | 0 | 0 | 0 | 0 | 0 | 0 | 0 | 0 | |
| Multiple births | 0 | 0 | 0 | 0 | 0 | 0 | 0 | 0 | |
| Major birth  defect | 0 | 0 | 0 | 0 | 0 | 0 | 0 | 0 | |
| Maternal | | | | | | | |  | |
| Non-white | 0 | 0 | 9 | 0 | 8 | 0 | 17 | 0 | |
| Prenatal care | 2 | 0 | 3 | 0 | 0 | 0 | 5 | 0 | |
| Chorioamnionitis  (from 2008) | 0 | 131 (2007 data) | 12 | 0 | 20 | 0 | 32 | 131 | |
| Maternal  hypertension,  chronic or  pregnancy induced (from 2008) | 0 | 131 (2007 data) | 4 | 0 | 14 | 0 | 18 | 131 | |
| Antenatal steroid exposure | 2 | 0 | 5 | 0 | 4 | 0 | 11 | 0 | |
| Vaginal delivery | 0 | 0 | 1 | 0 | 0 | 0 | 1 | 0 | |
| Antenatal magnesium sulphate (from 2012) | 0 (N/A) | 748 | 40 | 0 | 48 | 0 | 88 | 748 | |
| Surfactant during initial resuscitation | 2 | 0 | 0 | 0 | 1 | 0 | 3 |  | 0 |
| Surfactant at any time  (absolute) | 2 | 0 | 0 | 0 | 0 | 0 | 2 |  | 0 |
| Any non-invasive ventilation during resuscitation | 27 | 200 | 1 | 0 | 1 | 0 | 29 |  | 200 |
| Any non-invasive  ventilation after  resuscitation | 0 | 86 | 0 | 53 | 0 | 24 | 0 | | 163 |
| Any mechanical  ventilation during resuscitation | 0 | 0 | 0 | 0 | 1 | 0 | 1 | | 0 |
| Any mechanical ventilation after the initial  resuscitation | 0 | 4 | 0 | 2 | 0 | 3 | 0 | | 9 |

**Supplementary table 2: Summary of missing and non-applicable (N/A) data**

| **Co-factors** | **Mortality** | **Alive** | **p-value** |
| --- | --- | --- | --- |
| SGA | 60 (52.6) | 832 (37.7) | 0.001 |
| Out-born | 27 (23.7) | 499 (22.6) | 0.789 |
| Non white | 15 (13.3) | 193 (8.8) | 0.106 |
| Male | 63 (55.3) | 1132 (51.3) | 0.408 |
| Multiple births | 19 (16.7) | 640 (29.0) | 0.004 |
| Antenatal steroids | 90 (81.1) | 2002 (91.0) | <0.001 |
| Vaginal delivery | 34 (29.8) | 629 (28.5) | 0.762 |
| Gestation (weeks + days), median (IQR) | 30.1 (2.6) | 29.4 (2.4) | 0.001 |
| Admission  temperature, median (IQR) | 36.7 (1) | 36.8 (0.6) | 0.065 |
| **Co-factors** | **Severe CUSS abnormality** | **No severe CUSS abnormality** | **p-value** |
| SGA | 18 (16.7) | 821 (38.5) | <0.001 |
| Out-born | 32 (29.6) | 484 (22.7) | 0.094 |
| Non white | 8 (7.5) | 190 (9.0) | 0.597 |
| Male | 58 (53.7) | 1089 (51.0) | 0.588 |
| Multiple births | 23 (21.3) | 618 (29.0) | 0.086 |
| Antenatal steroids | 82 (75.9) | 1942 (91.5) | <0.001 |
| Vaginal delivery | 33 (30.6) | 603 (28.3) | 0.607 |
| Gestation (weeks + days), median (IQR) | 29.0 (1.43) | 30.1 (2.57) | <0.001 |
| Admission  temperature, median (IQR) | 36.9 (0.8) | 36.8 (0.6) | 0.048 |
| **Co-factors** | **ROP intervention** | **No ROP**  **intervention** | **p-value** |
| SGA | 10 (50.0) | 877 (38.3) | 0.285 |
| Out-born | 3 (15.0) | 522 (22.8) | 0.407 |
| Non white | 1 (5.0) | 206 (9.1) | 0.528 |
| Male | 9 (45.0) | 1179 (51.5) | 0.562 |
| Multiple births | 9 (45.0) | 649 (28.4) | 0.101 |
| Antenatal steroids | 18 (90.0) | 2067 (90.7) | 0.91 |
| Vaginal delivery | 2 (10.0) | 656 (28.7) | 0.066 |

| Gestation (weeks + days), median (IQR) | 29.3 (1.93) | 30.1 (2.57) | 0.013 |
| --- | --- | --- | --- |
| Admission  temperature, median (IQR) | 36.55 (0.7) | 36.8 (0.6) | 0.265 |
| **Co-factors** | **BPD** | **No BPD** | **p-value** |
| SGA | 110 (51.2) | 297 (43.4) | 0.047 |
| Out-born | 37 (17.2) | 83 (12.1) | 0.056 |
| Non white | 15 (7.1) | 78 (11.6) | 0.063 |
| Male | 135 (62.8) | 312 (45.6) | <0.001 |
| Multiple births | 60 (27.9) | 188 (27.5) | 0.904 |
| Antenatal steroids | 200 (93.0) | 623 (91.6) | 0.509 |
| Vaginal delivery | 42 (19.5) | 190 (27.8) | 0.016 |
| Gestation (weeks + days), median (IQR) | 29.6 (2.29) | 30.9 (2.86) | <0.001 |
| Admission  temperature, median (IQR) | 36.7 (0.5) | 36.7 (0.5) | 0.405 |
| **Co-factors** | **NEC** | **No NEC** | **p-value** |
| SGA | 29 (37.7) | 858 (38.4) | 0.897 |
| Out-born | 25 (32.5) | 501 (22.4) | 0.039 |
| Non white | 11 (14.3) | 195 (8.8) | 0.097 |
| Male | 35 (45.5) | 1154 (51.6) | 0.286 |
| Multiple births | 15 (19.5) | 643 (28.8) | 0.076 |
| Antenatal steroids | 70 (90.9) | 2019 (90.8) | 0.97 |
| Vaginal delivery | 19 (24.7) | 639 (28.6) | 0.453 |
| Gestation (weeks + days), median (IQR) | 29.6 (2.14) | 30.1 (2.57) | <0.001 |
| Admission  temperature, median (IQR) | 36.7 (0.7) | 36.8 (0.6) | 0.283 |
| **Co-factors** | **Any sepsis during stay** | **No sepsis during stay** | **p-value** |
| SGA | 103 (37.2) | 750 (38.5) | 0.682 |
| Out-born | 66 (23.8) | 451 (23.1) | 0.797 |
| Non white | 22 (8.0) | 177 (9.1) | 0.536 |
| Male | 156 (56.3) | 991 (50.8) | 0.087 |
| Multiple births | 68 (24.5) | 572 (29.3) | 0.1 |
| Antenatal steroids | 247 (90.1) | 1768 (91.0) | 0.629 |
| Vaginal delivery | 65 (23.6) | 563 (28.9) | 0.066 |
| Gestation (weeks + days), median (IQR) | 29.3 (2.29) | 30.1 (2.71) | <0.001 |
| Admission  temperature, median (IQR) | 36.7 (0.6) | 36.8 (0.6) | 0.169 |
| **Co-factors** | **Delivery Room Surfactant** | **No Delivery Room Surfactant** | **p-value** |
| SGA | 247 (29.1) | 644 (43.8) | <0.001 |
| Out-born | 206 (24.3) | 318 (21.6) | 0.14 |
| Non white | 71 (8.5) | 137 (9.4) | 0.465 |
| Male | 476 (56.1) | 717 (48.8) | <0.001 |
| Multiple births | 211 (24.9) | 447 (30.4) | 0.004 |
| Antenatal steroids | 750 (89.0) | 1340 (91.5) | 0.048 |
| Vaginal delivery | 250 (29.5) | 412 (28.0) | 0.445 |
| Gestation (weeks + days), median (IQR) | 29.1 (1.57) | 31.0 (2.57) | <0.001 |
| Admission  temperature, median (IQR) | 36.8 (0.7) | 36.7 (0.5) | 0.01 |
| **Co-factors** | **Delivery Room Ventilation** | **No Delivery Room Ventilation** | **p-value** |
| SGA | 261 (29.5) | 631 (44.0) | < 0.001 |
| Out-born | 220 (24.8) | 305 (21.3) | 0.046 |
| Non white | 76 (8.7) | 132 (9.3) | 0.622 |
| Male | 493 (55.6) | 701 (48.9) | 0.002 |
| Multiple births | 215 (24.3) | 444 (31.0) | <0.001 |
| Antenatal steroids | 779 (88.5) | 1313 (91.9) | 0.007 |
| Vaginal delivery | 266 (30.0) | 396 (27.6) | 0.216 |
| Gestation (weeks + days), median (IQR) | 29.3 (1.43) | 31.0 (2.57) | < 0.001 |
| Admission  temperature, median (IQR) | 36.8 (0.7) | 36.7 (0.5) | 0.039 |
| **Co-factors** | **Ventilation after Admission** | **No Ventilation after Admission** | **p-value** |
| SGA | 387 (30.4) | 500 (48.2) | < 0.001 |
| Out-born | 354 (27.8) | 172 (16.6) | < 0.001 |
| Non white | 92 (7.3) | 114 (11.1) | 0.002 |
| Male | 711 (55.8) | 478 (46.1) | <0.001 |
| Multiple births | 328 (25.7) | 330 (31.8) | 0.001 |
| Antenatal steroids | 1134 (89.5) | 955 (92.4) | 0.018 |
| Vaginal delivery | 367 (28.8) | 291 (28.0) | 0.674 |
| Gestation (weeks + days), median (IQR) | 29.6 (1.71) | 31.8 (2.71) | < 0.001 |
| Admission  temperature, median (IQR) | 36.8 (0.6) | 36.7 (0.5) | < 0.001 |

**Supplementary Table 3: Univariable analysis of co-factors. Co-factors with a p-value ≤ 0.1 were chosen for adjusted analysis.**

**References**

1. Jobe AH, Bancalari E. Bronchopulmonary dysplasia. *Am J Respir Crit Care Med* 2001;163(7):1723-9. doi: 10.1164/ajrccm.163.7.2011060
2. Kliegman RM, Walsh MC. Neonatal necrotizing enterocolitis: pathogenesis, classification, and spectrum of illness. *Current problems in pediatrics* 1987;17(4):213-88. [published Online First: 1987/04/01]
3. Papile LA, Burstein J, Burstein R, et al. Incidence and evolution of subependymal and intraventricular hemorrhage: a study of infants with birth weights less than 1,500 gm. *J Pediatr* 1978;92(4):529-34. doi: 10.1016/s0022-3476(78)80282-0
4. Chiang MF, Quinn GE, Fielder AR, et al. International Classification of Retinopathy of Prematurity, Third Edition. *Ophthalmology* 2021;128(10):e51-e68. doi:

10.1016/j.ophtha.2021.05.031
